# Supplementary material for: The Effect of Melasma on the Quality of Life in People with Darker Skin Types Living in Durban, South Africa
Source: Int J Environ Res Public Health. 2023 Nov 16;20(22):7068. doi: 10.3390/ijerph20227068 (PMC10671852; doi:10.3390/ijerph20227068)
Supplement: Supplementary file 1 [file ijerph-20-07068-s001.zip › File S1 Data Collection Survey Form.pdf]

Outline of the Procedures:
All willing participants who have completed the informed consent are encouraged to complete the questionnaire. Participation in the study will not interrupt participants as it will take 15 minutes to complete. Participants are free to withdraw from the study at any point, even while completing the questionnaire, if they feel any discomfort at any stage.

Risks or Discomforts to the participant:
There are no foreseeable risks or discomfort if you participate in this study.

Voluntary Participation:
You may withdraw from the study if any of the questions make you feel uncomfortable. There will be no adverse consequences if you choose to withdraw from the study.

Benefits:
You may find comfort in talking about your melasma experiences. Your full co-operation will add to the existing body of knowledge about melasma.

Reimbursements:
No money is offered for participation in this study.

Costs of the Study:
There are no costs associated with participating in this study.

Confidentiality:
The information given for the purpose of this research study will be kept confidential, all names will be replaced by codes and records will locked away. The key will be kept separate from the data. Only the investigators will have keys and access to the locked storage. Your eyes will be blocked out on photographs to protect your identity. The researcher is committed to ensuring your personal information is used appropriately, transparently and in accordance with the Protection of Personal information Act, 2013(POPIA). If

QuestionPro

requested, participants will be provided with summary feedback on the main findings of the research.

Sharing the Results:
Data collected from this research thesis will be shared with the research supervisors. This information will not be used for any advertising. Confidential information will not be shared. If requested, participants will be provided with summary feedback on the main findings of the research. The research findings will be presented to the Department of Dermatology and at conferences as part of the research component for the PhD degree.

Research-related Injury:
Not applicable to this study as it is an online questionnaire-based study.

Queries: who to contact:
The researcher, Nomakhosi Mpofana: mpofanancput@gmail.com / 0786087119

Supervisor, Prof Dlova: dlovan@ukznac.za/031 2604530
Co-supervisor, Prof Ahmed: mohammedam@cput.ac.za/021 4604217

For any further information regarding ethical issues, you can contact the Biomedical Research Ethics Committee on the contact details below:

Research Office, Westville Campus
Govan Mbeki Building
University of KwaZulu-Natal
Private Bag X 54001, Durban, 4000
KwaZulu-Natal, SOUTH AFRICA
Tel: 27 31 2602486 - Fax: 27 31 2604609
Email: BREC@ukzn.ac.za
Your assistance is highly appreciated
Nomakhosi Mpofana

Informed Consent

Statement of Agreement to Participate in the Research Study:

I hereby confirm that I have been informed by the researcher, Nomakhosi Mpofana, about the nature, conduct, benefits and risks of this study - Research Ethical Clearance Number: BREC/00002721/2021 I have also received, read and understood the written information (Participant Letter of Information) regarding the study.
I understand the purpose and the procedure of the study.
I understand that my participation is voluntary. I can refuse to participate at any point without giving any reason, and without penalty. I can ask to have all information obtained about me in the study to be returned to me, removed from the research records, or destroyed.
I understand that no individually-identifiable information about me, or provided by me during this study, will be shared with others without my written permission, except if it is necessary in order to protect my welfare (for example, if I were injured and needed physicians care) or if required by law.
I understand that there will be no cost to me for participation in this study and that I will not be compensated to participate in the study.
I declare that I take part in this study at my own risk and that UKZN, any of its workers or students are not responsible if anything should happen to me during the course of the study period.
I declare that I have read the details of the study and declare that I understand it. I have had the opportunity to discuss relevant aspects with the researcher and declare that I voluntarily participate in this research study.
If I have any further questions/concerns or queries related to the study, I understand that I may contact the researcher.
If I have any questions or concerns about my rights as a study participant, or if I am concerned about any aspect of the study or the researchers then I may contact the researcher.
I agree that the results of the study will be included in the PhD thesis and it may be written up in a scientific article. It may also be presented at a conference or workshop for educational purposes.

I hereby give consent to participate in the study.

I,................ hereby confirm that the above participant has been fully informed about the nature, conduct and risks of the above study.
Full name of the researcher : Date: 11/03/22 Signature:

* I agree to participate in the study

- ◯ Yes
- ◯ No

* Please add your initials

Please add your email address

Section A

Demographics (Please complete the following questions by filling in an answer or choosing an appropriate option)

* What is your age ?

- ◯ 18-24
- ◯ 25-34

- 35-44
- 45-54
- 55-64
- Above 64

* What is your level of education

- Below matric/grade 12
- Matric/grade 12
- Tertiary

* Occupation

- Indoor
- Outdoor
- Both
- NA

* Gender

- Female
- Male
- Non binary

* Ethnicity

- Black African

◯ Indian

◯ Coloured

◯ Other

* Do you have any children?

◯ Yes

◯ No

* If yes, how many children do you have?

◯ 1

◯ 2

◯ 3

◯ 4

◯ 5

◯ more than 5

Section B

Tell about your skin care routine

* List all skin care products that you use

☐ Makeup remover

☐ Cleanser

☐ Toner

QuestionPro

- [ ] Day cream/moisturiser
- [ ] Night cream/moisturiser
- [ ] Exfoliator
- [ ] Mask
- [ ] Serum
- [ ] Sun protection (SPF)

---

* Do you use sun protection?

- ◯ Yes
- ◯ No

---

* If yes, how often do you use your sun protection cream?

- ◯ Once a day
- ◯ Twice a day
- ◯ NA

---

* If yes, what is SPF of your sun protection cream?

- ◯ SPF 4
- ◯ SPF 15
- ◯ SPF 20
- ◯ SPF 30
- ◯ SPF 40-100
- ◯ Unknown

* If not using sun protection cream, please explain why?

* Have you ever had any reaction on cosmetics or sun protection creams?

- Yes
- No

* If yes, please specify

- Cosmetics
- Sun protection cream
- NA

Factors related to melasma

Tell more about your skin condition

* Have you consulted the doctor/therapist/herbalist for melasma or ill health in the last three months?

- Yes
- No

* List all your current medication, vitamins or supplements

QuestionPro

* Are you familiar with the word "melasma"?

- ◯ Yes
- ◯ No
- ◯ Not sure

* Do you suffer from melasma ?

- ◯ Yes
- ◯ No
- ◯ Not sure

* How long have you suffered from melasma for?

- ◯ 6 months
- ◯ 1 year
- ◯ 2 years
- ◯ 5 years
- ◯ 10 years
- ◯ Longer

* Where on your face is melasma

- ☐ Forehead
- ☐ Cheeks
- ☐ Jawline
- ☐ Nose

☐ Sides of the face

☐ All of the above

☐ Don't know

* Do you use any treatment for melasma?

○ Yes

○ No

* If yes, how long have you been using the treatment for?

○ Less than 3 months

○ Less than 6 months

○ More than a year

○ More than two years

○ More than 5 years

* List all creams, tablets, non-medical or traditional treatments you use for melasma

* Is there any improvement since the use of the treatment?

○ Yes

○ No

○ Not sure

What difference have you observed since the treatment of melasma?

* What difference have you observed since the treatment of melasma?

- Lightening of melasma
- Darkening of melasma
- Worsening of melasma
- No difference

* Have you used any other non-medical treatment or traditional plants for melasma?

- Yes
- No

* If yes, please provide the name of the non-medical home made intervention

* How does this home made treatment intervention work?

* Are there any family members who suffer melasma?

- Yes
- No

QuestionPro

* If yes, who?

- [ ] Mother
- [ ] Father
- [ ] Sister
- [ ] Brother
- [ ] Aunt
- [ ] Uncle
- [ ] Cousin

* What do you think triggers your melasma

- [ ] Sun exposure
- [ ] Heat
- [ ] Cosmetics
- [ ] Medication
- [ ] Occupation
- [ ] Stress
- [ ] Pill
- [ ] Other

* Are you involved in any outdoor sport?

- ( ) Yes
- ( ) No

* If yes, how often

If yes, how often

- Once a week
- Twice a week
- Daily
- Not involved

* Have you reached menopause?

- Yes
- No
- Do not know
- N/A (non-menopausal participants

* If yes, a what age?

- 40
- 45
- 50
- 55
- N/A (non-menopausal participants)
- Not sure

* If yes, has your melasma improved after menopause or got worse

- Improved
- Worsened

- ○ Remained the same
- ○ N/A (non-menopausal participants)
- ○ Not sure

* Are you using any hormonal replacement therapy (HRT)?

- ○ Yes
- ○ No
- ○ Not sure
- ○ N/A (non-menopausal participants)

* If yes, What has been the effect of HRT on melasma

- ○ Improved
- ○ Same
- ○ Worse
- ○ N/A (non-menopausal participants)
- ○ Don't know

MELASQoL

Tell about the effect of melasma on your life

* On a Likert scale of 1 (not bothered at all) to 7 (bothered at all times), rate how you feel about yourself based on the following points:

	1 (Not bothered at all)	2 (Not really bothered)	3 (Not bothered sometimes)	4 (Neutral)	5 (Bothered sometimes)	6 (Bothered most of the time)	7 (Bothered all the time)
The appearance of your skin condition	☐	☐	☐	☐	☐	☐	☐
Frustration about your skin condition	☐	☐	☐	☐	☐	☐	☐
Embarrassment about your skin condition	☐	☐	☐	☐	☐	☐	☐
Feeling depressed about your skin condition	☐	☐	☐	☐	☐	☐	☐
Effects of your skin condition on your interaction with other people (family, children, friends, work colleagues)	☐	☐	☐	☐	☐	☐	☐
Effects of your skin condition on your desire to be with people	☐	☐	☐	☐	☐	☐	☐
Your skin condition making it hard to show affection	☐	☐	☐	☐	☐	☐	☐
Skin discolouration making you feel unattractive to others	☐	☐	☐	☐	☐	☐	☐
Skin discolouration making you feel less vital or productive	☐	☐	☐	☐	☐	☐	☐
Skin condition/discolouration affecting your sense of freedom	☐	☐	☐	☐	☐	☐	☐
