# Supplementary material for: The Effect of Melasma on the Quality of Life in People with Darker Skin Types Living in Durban, South Africa
Source: Int J Environ Res Public Health. 2023 Nov 16;20(22):7068. doi: 10.3390/ijerph20227068 (PMC10671852; doi:10.3390/ijerph20227068)
Supplement: Supplementary file 1 [file ijerph-20-07068-s001.zip › File S2 STROBE checklist.pdf]

File S2 STROBE checklist

	Item No	Recommendation
**Title and abstract**	1	(*a*) Indicate the study's design with a commonly used term in the title or the abstract<br><br>Cross sectional study<br>The Effect of Melasma on the Quality of Life in People with Darker Skin Types Living in Durban, South Africa
		(*b*) Provide in the abstract an informative and balanced summary of what was done and what was found<br><br>Melasma is a common skin disorder of acquired hyperpigmentation that appears commonly on the face. Although asymptomatic, melasma causes psychosocial and emotional distress. This study aimed to assess melasma's severity on people with darker skin types, evaluate the effects of melasma on the quality of life (QoL), and establish QoL predictors in affected individuals. This was a cross-sectional analytic study that enrolled 150 patients from three private dermatology clinics in Durban, South Africa who were diagnosed with melasma. The severity of melasma, and QoL were measured using a melasma area and severity index (MASI) score, and melasma quality of life scale (MELASQoL) respectively. The associations among factors and QoL were explored using multivariable methods and stepwise regression analysis. P-values less than 0.05 were considered significant. Enrolled patients were predominantly females (95%), of which 76% were of black African ethnicity, 9% were of Indian ethnicity, and 15% had mixed ancestry, with an average age of 47.30 years. Family history revealed that 61% had no prior melasma cases, while 39% had affected relatives, most commonly mothers (41%). The cheeks were the most common site for melasma. MASI score of Masi ($\beta = 0.209$, $t = 2.628$, $p < .001$), involvement of cheeks ($\beta = -0.268$, $t = -3.405$, $p < .001$), level of education ($\beta = -0.159$, $t = -2.029$, $p = .044$) and being menopausal ($\beta = -0.161$, $t = -2.027$, $p = .045$) were found to be predictors of QoL. A regression model was created to forecast MELASQoL using these four predictors. This equation's significance lies in its ability to enable remote assessment of MELASQoL based on these four variables. It offers a valuable tool for researchers and medical professionals to quantitatively and objectively evaluate the impact of melasma on an individual's quality of life.
**Introduction**		
Background/rationale	2	Explain the scientific background and rationale for the investigation being reported<br><br>The most common complaints dermatologists deal with are premature aging, acne, and pigmentary disorders including melasma. Melasma is a common dyschromia, mainly found in women between Fitzpatrick skin types IV-VI. Melasma prevalence varies across the globe ranging from 1% in the population as a whole to 9 - 50% in populations at higher risk. This broad variance in prevalence has been attributed primarily to differences in ethnicity and levels of sun exposure among population groups living in different geographic regions.<br>Melasma is often associated with a variety of factors such as sun exposure, genetics, sex steroids, drugs, or cosmetics. Due to this complex pathogenesis, melasma is difficult to target and likely to recur after treatment. Oral therapies (tranexamic acid, glutathione), procedural interventions (chemical peels, microneedling, lasers, and

		lights), and topical therapies (tretinoin, hydroquinone, triple combination) are helpful, however, they are not suitable for all skin types due to undesired side effects, and suboptimal results, more especially when dealing with darker skin types (Fitzpatrick skin types IV-VI).<br>Although asymptomatic, melasma as a facial disorder affects the appearance of facial skin aesthetically and can reduce a person's confidence, resulting in a low quality of life for the patient. The burden of this skin disease is an intricate concept that includes the emotional, social interactions, and economic impact of skin disease on individuals, their families, and society. Hence, personal and socioeconomic factors have been shown to have an impact on health-related QoL<br>The melasma quality of life scale (MELASQoL) is one of the validated dermatology-specific instruments used to assess the impact of melasma on health-related quality of life (HRQoL). However, no evidence shows it has been fully explored in clinical practice in South Africa. With an emphasis on people with darker skin types, the aim of this study is to comprehensively understand melasma by answering the few research questions as follows:<br>1.What are the key factors influencing the severity of melasma in individuals with darker skin types (Fitzpatrick skin types IV-VI)?<br>2.How does melasma impact the QoL of individuals with darker skin types?<br>3.Can a predictive model be developed to assess the impact of melasma on the quality of life?<br>Answering these research questions could help the treatment intervention to be directed more appropriately
Objectives	3	State specific objectives, including any prespecified hypotheses<br>With an emphasis on people with darker skin types, the aim of this study is to comprehensively understand melasma by achieving the following objectives:<br>1.Assess the severity of melasma,<br>2.Evaluate the impact of melasma on the QoL of affected patients,<br>3.Identify predictors of QoL through stepwise regression analysis.<br>The research questions that guided the study were as follows:<br>1.What are the key factors influencing the severity of melasma in individuals with darker skin types (Fitzpatrick skin types IV-VI)?<br>2.How does melasma impact the QoL of individuals with darker skin types?<br>3.Can a predictive model be developed to assess the impact of melasma on the quality of life?
**Methods**		
Study design	4	Present key elements of study design early in the paper<br><br>We conducted a cross-sectional study from three private dermatology clinics in Durban. Descriptive statistics on continuous data were conducted whilst frequencies were reported for categorical variables. A Pearson correlation matrix was used to establish the presence of multicollinearity between predictor variables. A stepwise regression was performed to establish statistically significant predictors of QoL. The stepwise regression algorithm identifies predictor variables with p-values for the F-statistic $\leq .050$ are considered for inclusion in the model, while variables with p-values $\geq .100$ are removed from the model. Before the stepwise regression, the variables were evaluated for the presence of multicollinearity.
Setting	5	Describe the setting, locations, and relevant dates, including periods of recruitment,

		exposure, follow-up, and data collection<br>Data were collected between March and December 2022. All patients who had melasma based in the dermatologist diagnosis had further detailed clinical examination by the same dermatologist. The MELASQoL questionnaire was administered to respondents to measure their QoL. The severity of melasma was graded based on MASI.
Participants	6	(*a*) Give the eligibility criteria, and the sources and methods of selection of participants<br>We enrolled a total of 150 patients from an existing data-base. The inclusion criteria included all patients who are classified as darker skinned types (Fitzpatrick skin types IV-VI), both male and female, who have been diagnosed with either epidermal, dermal or mixed facial melasma. Patients who were older than 18 years and provided consent were enrolled in the study. The exclusion criteria included patients who were diagnosed with extra facial melasma, as well as other disorders of hypermelanosis, other melasma. Patients with lighter skin types (Fitzpatrick skin types I-III) were exclude from the study as well as all vulnerable people and minors. The survey was administered to patients in English, either online or face-to-face by a trained multilingual interviewer
Variables	7	Clearly define all outcomes, exposures, predictors, potential confounders, and effect modifiers. Give diagnostic criteria, if applicable<br><br>Twenty-eight predictor variables were assessed. This consisted of a number of binary (dummy) and continuous variables. They are clearly defined and relevance explained. Multicollinearity and Variance Inflation Factor (VIF) were used for determining the presence of multi-collinearity between predictors. Stepwise regression analysis was performed to leave only statistically significant predictor variables.
Data sources/ measurement	8*	For each variable of interest, give sources of data and details of methods of assessment (measurement). Describe comparability of assessment methods if there is more than one group<br><br>Sources of data are primary and have been collected from a survey instrument
Bias	9	Describe any efforts to address potential sources of bias<br><br>To remove any potential sources of bias, a rigorous sampling protocol was employed and all variables chosen based on previous peer reviewed literature. All methods have been transparently been reported on.
Study size	10	Explain how the study size was arrived at<br><br>The sample size was determined based on previous studies that have used similar methodologies to assess melasma severity and its impact on QoL. Our current study mirrors Dlova et al, 2019 research, in which they sought to use their prevalence data to enhance dermatological services in KwaZulu-Natal and South Africa as a whole. This alignment with their objectives led us to choose the same sampling location.
Quantitative variables	11	Explain how quantitative variables were handled in the analyses. If applicable, describe which groupings were chosen and why<br><br>Quantitative variables were descriptively assessed for their locality (i.e. mean) and dispersion (i.e. standard deviation). Their distributions (i.e. the Kolmogorov–Smirnov

		test and the Shapiro–Wilk test for normality) were not assessed and is no need for an MLR.
Statistical methods	12	Describe all statistical methods, including those used to control for confounding<br><br>Test for multicollinearity using VIF test and correlation matrix and stepwise multiple variable regression
		(*b*) Describe any methods used to examine subgroups and interactions<br>Subgroups were not segmented out for multiple groups analysis (MGA).
		(*c*) Explain how missing data were addressed<br>The dataset contained no missing data was found
		(*d*) If applicable, describe analytical methods taking account of sampling strategy<br>The sampling strategy was purposive were qualified dermatologists assessed patients for the study.
		(*e*) Describe any sensitivity analyses<br>None assessed.
**Results**		
Participants	13*	(a) Report numbers of individuals at each stage of study—eg numbers potentially eligible, examined for eligibility, confirmed eligible, included in the study, completing follow-up, and analysed<br>N =150
		(b) Give reasons for non-participation at each stage<br>N/A
		(c) Consider use of a flow diagram<br>N/A
Descriptive data	14*	(a) Give characteristics of study participants (eg demographic, clinical, social) and information on exposures and potential confounders<br><br>Enrolled patients were predominantly females (95%), of which 76% were of black African ethnicity, 9% were of Indian ethnicity, and 15% had mixed ancestry, with an average age of 47.30 years. Family history revealed that 61% had no prior melasma cases, while 39% had affected relatives, most commonly mothers (41%). The cheeks were the most common site for melasma
		(b) Indicate number of participants with missing data for each variable of interest<br>N/A
Outcome data	15*	Report numbers of outcome events or summary measures<br>N/A
Main results	16	(*a*) Give unadjusted estimates and, if applicable, confounder-adjusted estimates and their precision (eg, 95% confidence interval). Make clear which confounders were adjusted for and why they were included<br>No confounder variables were assumed, nor identified in the literature. This was an exploratory study to focus on the impact of predictor variables on QoL.
		(*b*) Report category boundaries when continuous variables were categorized<br>N/A
		(*c*) If relevant, consider translating estimates of relative risk into absolute risk for a meaningful time period<br>N/A

Other analyses	17	Report other analyses done—eg analyses of subgroups and interactions, and sensitivity analyses<br>N/A
**Discussion**		
Key results	18	Summarise key results with reference to study objectives<br><br>Melasma has a significant impact on a patient's quality of life (QoL). In this study, we found that impairment of quality of life is greater irrespective of the underlying melasma conditions. Even when melasma is not severe, it can cause emotional stress, potentially reducing patients' quality of life. Through the stepwise regression model, we distilled 4 key predictor variables out of 28 and developed a regression model to predict MELASQoL given these four predictors. The significance of the equation can allow e.g. remote scoring of MELASQoL based on the four variables, which could help customize the treatment intervention based on the forecasted score
Limitations	19	Discuss limitations of the study, taking into account sources of potential bias or imprecision. Discuss both direction and magnitude of any potential bias<br><br>We acknowledge that the fact that the data may not necessarily be representative of the complete health care population of study area, however, this region does represent the most populous region for darker skin type women in South Africa, steeped in cultural norms and practices thereby providing a good test case for future research. The selected participants' experiences with melasma, may not fully represent the diversity of views and the QoL in the Durban community as a whole as only darker skin type people were enrolled in the study, additionally a small number of men enrolled in the study were not a true representation. Also, people who reside in the rural areas could express different results due dissimilar lifestyle habits.
Interpretation	20	Give a cautious overall interpretation of results considering objectives, limitations, multiplicity of analyses, results from similar studies, and other relevant evidence<br><br>Melasma has a significant impact on a patient's quality of life. Even when melasma is not severe, it can cause emotional stress, potentially reducing patients' quality of life. Through the stepwise regression model, we distilled 4 key predictor variables out of 28 and developed a regression model to predict MELASQoL given these four predictors. The significance of the equation can allow e.g. remote scoring of MELASQoL based on the four variables, which could help customize the treatment intervention based on the forecasted score.
Generalisability	21	Discuss the generalisability (external validity) of the study results<br><br>The study findings could help inform treatment protocol based on individual indicated quality of life. The developed regression model could be a useful tool to help with remote scoring of MELASQoL.
**Other information**		
Funding	22	Give the source of funding and the role of the funders for the present study and, if applicable, for the original study on which the present article is based<br><br>This work is based on research supported in part by the National Research Foundation of South Africa (Grant Number: 138179), the Department of Science and Innovation

	(DSI) "Cosmeceutical concepts and product development" project; the HWSETA bursary, and the Durban University of Technology Seed Funding grant

*Give information separately for exposed and unexposed groups.

Note: An Explanation and Elaboration article discusses each checklist item and gives methodological background and published examples of transparent reporting. The STROBE checklist is best used in conjunction with this article (freely available on the Web sites of PLoS Medicine at http://www.plosmedicine.org/, Annals of Internal Medicine at http://www.annals.org/, and Epidemiology at http://www.epidem.com/). Information on the STROBE Initiative is available at www.strobe-statement.org.
